# Dissecting the Structural Organization, Recruitment and Activation Mechanisms of Centrosomal γ‐TuRCs

**DOI:** 10.1002/cm.22040

**Published:** 2025-05-12

**Authors:** Florian W. Hofer, Martin Würtz, Qi Gao, Bram J. A. Vermeulen, Elmar Schiebel, Stefan Pfeffer

**Affiliations:** ^1^ Center for Molecular Biology of Heidelberg University (ZMBH) Heidelberg Germany; ^2^ European Molecular Biology Laboratory (EMBL) Heidelberg Germany

**Keywords:** centrosomes, cryo‐electron tomography, gamma‐tubulin ring complex, microtubule nucleation

## Abstract

Visualizing human centrosomes using cryo‐electron tomography revealed the native structure and molecular organization of γ‐tubulin ring complexes (γ‐TuRCs). γ‐TuRCs localized to two distinct centrosomal pools, one in the pericentriolar material (PCM) and another in the centriole lumen, which is released during mitosis. All detected γ‐TuRCs were associated with the tetrameric adaptor protein NEDD1. Within the PCM, binding to the centrosomin (CM1) motif of the microcephaly protein CDK5RAP2 in different patterns correlates with conformational changes of γ‐TuRCs. In the centriole lumen, the augmin complex anchors γ‐TuRCs to the inner scaffold. These observations provide key insights into how the structural organization of γ‐TuRCs and regulatory factors collectively govern the spatial and temporal control of microtubule nucleation in centrosomes.

The γ‐tubulin ring complex (γ‐TuRC) acts as a structural scaffold for the de novo formation of microtubules (MTs) from α/β‐tubulin dimers (Zheng et al. 1995), and localizes to microtubule organization centers (MTOCs), such as the mammalian centrosome (Mitchison and Kirschner 1984). MTs play essential roles in intracellular transport, motility, and cell division, and spatiotemporal control of their nucleation is therefore vital for cellular function (Prosser and Pelletier 2017). Cryo‐EM analysis of isolated vertebrate γ‐TuRCs revealed that the complex consists of 14 spokes, each containing one of five paralogous γ‐tubulin complex proteins (GCP2–6) bound to one molecule of γ‐tubulin, which serves as a binding site for α/β‐tubulin dimers during MT nucleation (Consolati et al. 2020; Liu et al. 2020; Wieczorek, Urnavicius, et al. 2020). The N‐terminal extensions of most GCP variants form intercalated folds with the structurally related mitotic spindle‐organizing proteins MZT1 (GCP3, GCP5, and GCP6) and MZT2 (GCP2), termed MZT1 and MZT2 modules, respectively (Wieczorek, Urnavicius, et al. 2020; Wieczorek, Huang, et al. 2020; Würtz et al. 2022; Xu et al. 2024; Serna et al. 2024; Zimmermann et al. 2020).

Interestingly, isolated vertebrate γ‐TuRCs were observed in an asymmetric ‘open’ conformation not matching MT geometry, explaining why they are poor templates for MT nucleation (Consolati et al. 2020; Liu et al. 2020; Wieczorek, Urnavicius, et al. 2020; Würtz et al. 2022; Xu et al. 2024; Serna et al. 2024). Consistently, recent cryo‐EM studies of MT‐capping γ‐TuRCs revealed partially (Vermeulen et al. 2024) or fully ‘closed’ γ‐TuRC conformations (Dendooven et al. 2024; Aher et al. 2024; Brito et al. 2024), which indicated that MT nucleation activity of γ‐TuRCs could be regulated by factors impacting on γ‐TuRC conformation. Indeed, γ‐TuRC function requires a complex interplay of various recruiting and activation factors, most prominently the universal adaptor protein NEDD1, the MT‐branching factor augmin, and the γ‐tubulin complex receptor protein CDK5RAP2, which interacts with the γ‐TuRC via its conserved CM1 motif and thereby elevates its MT nucleation activity (Haren et al. 2006; Fong et al. 2008; Gavilan et al. 2018; Lüders et al. 2006; Goshima et al. 2008; Petry et al. 2013; Zupa et al. 2022).

Aiming to investigate the structural basis for the complex interplay of these factors in a physiological context, a recent study (Gao et al. 2025) used cryo‐electron tomography, complemented by functional experiments, to examine the structure and conformational landscape of γ‐TuRCs in purified centrosomes and intact human cells. In this study, γ‐TuRCs were observed to localize to two centrosomal compartments: one dispersed pool within the pericentriolar material (PCM) and a second more condensed pool in the centriole lumen, in line with previous expansion microscopy analysis (Figure 1A).

**FIGURE 1:**
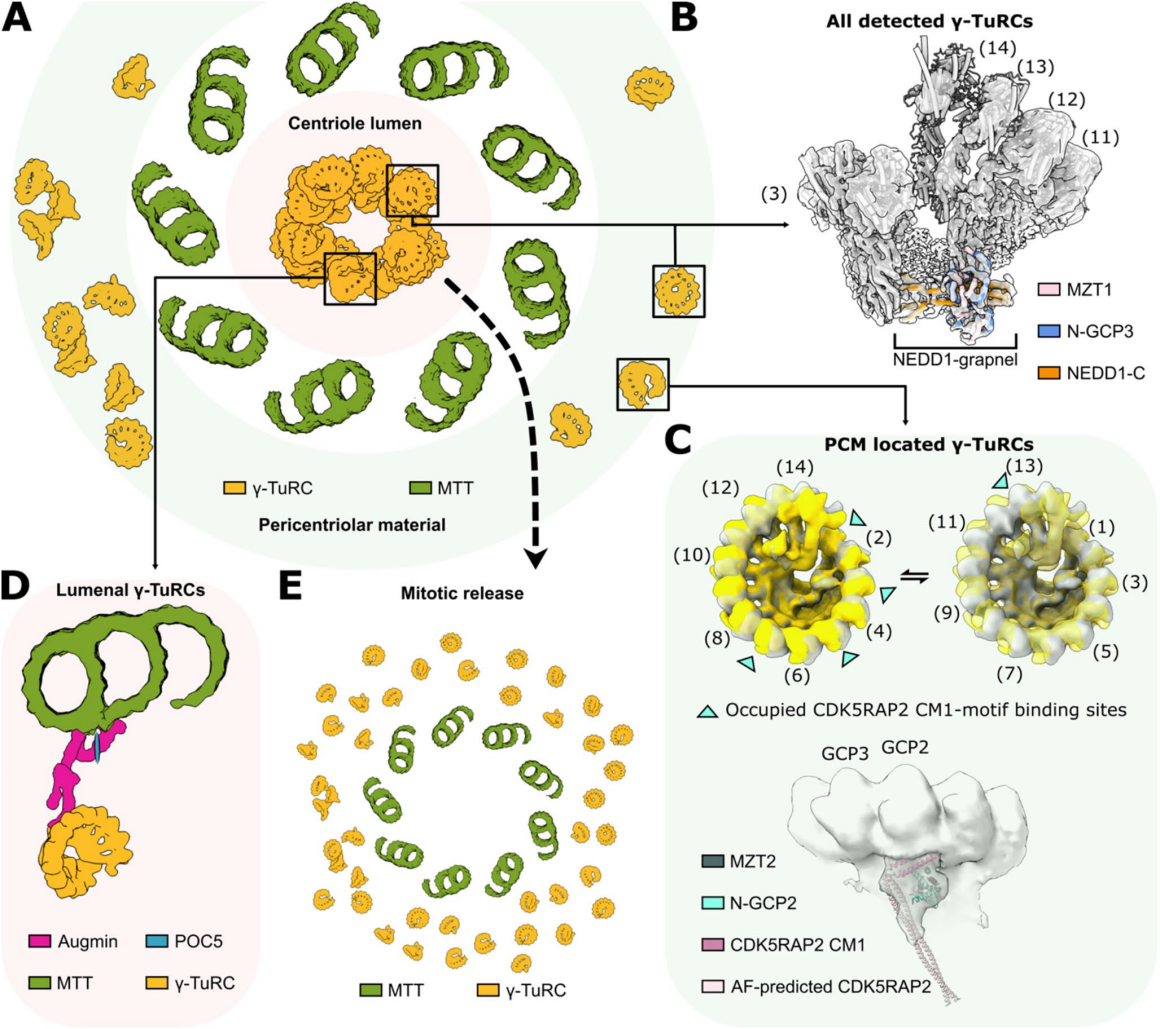
Sub‐centrosomal organization of γ‐TuRCs. (A) Schematic representation of γ‐TuRC localization in human centrosomes, illustrating pools within the centriole lumen (red shade) and in the PCM (green shade). (B) Cryo‐EM density of purified *Xenopus laevis* γ‐TuRC with tetrameric NEDD1/MZT1 module bound. NEDD1 was observed on all centrosomal γ‐TuRCs. Numbering of GCP‐γ‐tubulin spokes are indicated. Colouring as indicated. (C) Top: CDK5RAP2 CM1 motif binding in two different patterns is accompanied with conformational changes of the γ‐TuRC. Bottom: Cryo‐EM density of GCP2/GCP3 units with bound CM1 motif. Colouring as indicated. (D) Model depicting how the centrosomal pool of γ‐TuRCs is coordinated by POC5‐augmin–NEDD1 interactions. (E) During mitosis, this pool of γ‐TuRCs is released into the cytoplasm.

Regardless of their sub‐centrosomal localization, γ‐TuRCs were stoichiometrically associated with a grapnel‐shaped, fourfold symmetrical density segment bound to the narrow end of the γ‐TuRC spiral. Complementary high‐resolution cryo‐EM analysis of γ‐TuRCs purified from *Xenopus laevis* egg extracts, in combination with AlphaFold‐based structure predictions (Jumper et al. 2021) and interaction analysis, identified this density segment as the previously unknown binding site of the main adaptor protein NEDD1. Specifically, the grapnel shaped structure comprises a tetrameric coiled‐coil formed by the C‐terminal helices of four copies of NEDD1, each interacting with one GCP3‐derived MZT1 module (Figure 1B). In this configuration, the tetramerized C‐terminal helices of NEDD1 mediate interaction with the γ‐TuRC, while the flexibly linked beta‐propeller domains at the NEDD1 N‐terminus may allow the NEDD1/γ‐TuRC complex to be anchored and oriented in various subcellular compartments, in line with NEDD1's essential role as universal recruiting module for different MT nucleation pathways.

One such subcellular compartment is the PCM. Previous experiments suggested that γ‐TuRCs are recruited to the PCM by an interaction between NEDD1 (Haren et al. 2006; Lüders et al. 2006) and the PCM component CEP192 (Chinen et al. 2021; O'Rourke et al. 2014; Zhu et al. 2008; Gomez‐ Ferreria et al. 2007). Consistently, PCM‐localized γ‐TuRCs were observed stoichiometrically associated with NEDD1. The second main interactor and activator of PCM‐localized γ‐TuRCs is the γ‐tubulin complex receptor protein CDK5RAP2. Notably, CDK5RAP2 and NEDD1 were simultaneously bound to γ‐TuRCs, suggesting that these two key factors may cooperate in localizing and regulating PCM‐located γ‐TuRCs. In previous cryo‐EM studies using purified components, the binding site of the conserved CDK5RAP2 CM1 motif was identified to be located on GCP2 subunits of the complex (Xu et al. 2024; Serna et al. 2024). In these studies, CM1 motif binding on up to all five GCP2 subunits was observed to induce an allosteric conformational change towards a more MT‐compatible γ‐TuRC conformation, providing one possible explanation for the activating effect of the CM1 motif on MT nucleation. A similar CM1 motif‐dependent conformational change could also be observed for native PCM‐localized γ‐TuRCs, supporting the model of conformational activation (Figure 1C). However, while GCP2 subunits were indiscriminately decorated when using purified γ‐TuRCs and recombinant CM1 motif, two mutually exclusive patterns of native CDK5RAP2 CM1 motif binding, differentially affecting the γ‐TuRC conformation, were observed in native PCM‐localized γ‐TuRCs. This suggests that additional layers of regulation may participate in fine‐tuning the conformational landscape and interaction network of the γ‐TuRC in the native centrosomal context.

The second major pool of centrosomal γ‐TuRCs was observed in the centriole lumen. γ‐TuRC localization to the centriole lumen was reported to require the centriolar inner scaffold component POC5 and the MT‐branching factor Augmin (Schweizer et al. 2021). Integrated information from cryo‐electron tomography, MINFLUX nanoscopy (Gwosch et al. 2020; Schmidt et al. 2021) and AlphaFold‐guided reconstitution experiments supported a model in which Augmin is radially oriented in the centriole lumen, one end of the elongated complex interacting with POC5 at the centriolar inner scaffold, the other end coordinating γ‐TuRCs in the centriole lumen via interactions with NEDD1 (Figure 1D). By immunofluorescence imaging, it was observed that the lumenal pools of Augmin, NEDD1 and γ‐TuRC were released from the centriole lumen at the onset of mitosis in a PLK1‐dependent manner (Figure 1E) and thereby may contribute to spindle formation by rapidly enhancing the MT‐nucleation capacity during mitosis.

In summary, exploring the structure, molecular organization and function of γ‐TuRCs in the context of native centrosomes provided an important advance for detailed mechanistic understanding of how γ‐TuRC targeting and activation factors, including NEDD1 and CDK5RAP2, act in concert to control the spatiotemporal regulation of MT nucleation. Identification and structural analysis of MT‐capping γ‐TuRCs in the cellular context will provide additional important information on how MTs are anchored and turned over at centrosomes.

## Conflicts of Interest

The authors declare no conflicts of interest.
